# CpG-ODN Signaling via Dendritic Cells-Expressing MyD88, but Not IL-10, Inhibits Allergic Sensitization

**DOI:** 10.3390/vaccines9070743

**Published:** 2021-07-05

**Authors:** Ricardo Wesley Alberca, Eliane Gomes, Momtchilo Russo

**Affiliations:** 1 Institute of Biomedical Sciences, Department of Immunology, University of Sao Paulo, São Paulo 05508-000, Brazil; ricardowesley@usp.br (R.W.A.); melloeag@usp.br (E.G.); 2 Laboratory of Dermatology and Immunodeficiencies (LIM-56), Department of Dermatology, Institute of Tropical Medicine, Faculdade de Medicina FMUSP, Universidade de Sao Paulo, Sao Paulo 01246-903, Brazil

**Keywords:** allergic sensitization, eosinophils, dendritic cells, CpG-ODN, toll-like receptor, MyD88, IL-10

## Abstract

Allergen-specific T helper (Th)2 cells orchestrate upon allergen challenge the development of allergic eosinophilic lung inflammation. Sensitization with alum adjuvant, a type 2 adjuvant, has been used extensively in animal models of allergic lung disease. In contrast, type 1 adjuvants like CpG-ODN, a synthetic toll-like receptor 9 agonist, inhibit the development of Th2 immunity. CpG-ODN induce type 1 and suppressive cytokines that influence Th2 cell differentiation. Here, we investigated the immune modulatory effect of CpG-ODN on allergic sensitization to OVA with alum focusing on dendritic cells (DCs) expressing the MyD88 molecule and the suppressive IL-10 cytokine. Using mice with specific cell deletion of MyD88 molecule, we showed that CpG-ODN suppressed allergic sensitization and consequent lung allergic inflammation signaling through the MyD88 pathway on dendritic cells, but not on B-cells. This inhibition was associated with an increased production of IL-10 in the bronchoalveolar lavage fluid. Sensitization to OVA with CpG-ODN of IL-10-deficient, but not wild-type mice, induced a shift towards Th1 pattern of inflammation. Employing bone marrow-derived dendritic cells (BM-DCs) pulsed with OVA for sensitizations with or without CpG-ODN, we showed that IL-10 is dispensable for the inhibition of allergic lung Th2 responses by CpG-ODN. Moreover, the lack of IL-10 on DCs was not sufficient for the CpG-ODN-induced immune-deviation towards a Th1 pattern. Accordingly, we confirmed directly the role of MyD88 pathway on DCs in the inhibition of allergic sensitization.

## 1. Introduction

Prophylactic and therapeutic immune interventions have been investigated in experimental models of allergic lung inflammation. The ovalbumin (OVA) allergen and the aluminum hydroxide gel (alum) adjuvant have been extensively used to induce Th2-cell-dominated immune responses in the lung [1]. In contrast, Toll-like receptor (TLR) agonists have been used as adjuvants to downmodulate the Th2-dominated immune response [1,2]. Among the TLR agonists used experimentally, a TLR4 agonist composed of monophosphoryl lipid A (MPLA), and a TLR9 agonist, composed of oligodeoxynucleotides (ODN) containing CpG motifs (CpG-ODN), have been extensively used in clinical trials [3,4,5] and a formulation with CpG-ODN has been approved for human use [6].

Dendritic cells (DCs) are key cells that orchestrate and shape the adaptive immune response [7,8]. Depending on the scenario, DCs elicit different immune responses by activating effector T cells or inducing T-cell tolerance [9,10]. Therefore, DCs play a role in the induction and also in abrogation of allergic immune responses [11]. Since DCs stimulation with TLR agonists can induce both the production of pro-inflammatory cytokines like Th-1-inducing IL-12 and the anti-inflammatory molecules like IL-10 [12,13], the combination of CpG-ODN with anti-IL-10 receptor monoclonal antibody have been successfully used to improve DC-induced anti-tumor T-cell responses [14].

We have previously shown that the addition of CpG-ODN to alum adjuvant prevents the development of an allergen-specific eosinophilic lung inflammation, mucus formation and type-2 cytokine production without inducing in the lung, a deviation towards a Th1-dominated inflammation [15]. Importantly, the inhibition of allergic lung inflammation was not restricted to OVA [15], as it was also obtained using the extract of *Blomia tropicalis*, a relevant house dust mite in Brazil [16]. CpG-ODN was shown to be the most effective TLR agonist to prevent the development of Th2 response [15] and IL-10 production was required to shape CpG-ODN adjuvant activity, since in IL-10-deficient mice, sensitization to OVA with alum and CpG-ODN resulted in OVA-induced Th1-dominated immune responses with increased IFN-γ production and neutrophilic lung inflammation [15,16]. Recently, we demonstrated that downregulation allergen-specific immunoglobulin IgE and upregulation of IgG2c by sensitization with formulation containing CpG-ODN was dependent of MyD88 signaling on CD11c-positive putative DCs but not on CD19-positive B cells [17].

Here, using a mouse OVA model of allergic lung inflammation, we investigated the role of MyD88 or IL-10 expression on DCs in the immunomodulatory effect of CpG-ODN. For this, we used two types of approaches of in vivo sensitizations: one with alum adjuvant sensitization with or without CpG-ODN in mice with total MyD88-deficiency mice, or mice with specific deletion of MyD88 molecule on CD11c-positive DCs or CD-19-positive B cells, and the other in mice sensitized with bone marrow-derived dendritic cells (BM-DCs) lacking MyD88 molecule or IL-10 primed with OVA or OVA and CpG-ODN.

We found that the expression of MyD88 on DCs is essential for the anti-Th2 effect of CpG-ODN, while IL-10 expression on DCs is not necessary for this effect and its absence in DCs does not induce immune deviation towards Th1 immunity.

## 2. Materials and Methods

### 2.1. Mice

Six-to-eight-week-old female C57BL/6 mice (WT), IL-10-deficient (IL-10^−/−^), MyD88-deficient (MyD88^−/−^), Myd88 fl/fl and mice expressing the recombinase Cre under the control of the Itgax (CD11c) or CD19 promoter were originally purchased from Jackson Laboratories (Bar Harbor, ME) [12] and kept at the Institute of Biomedical Sciences of the University of Sao Paulo (ICB IV-USP) at a specific pathogen-free reproduction facility. CD19MyD88^−/−^ (B-MyD88^−/−^) or CD11cMyD88^−/−^ (DC-MyD88^−/−^) mice with specific deletion of MyD88 on B cells or DCs were generated by breeding Myd88fl/fl mice with CD11c-Cre (DC-MyD88^−/−^) or CD19-Cre (B-MyD88^−/−^). All mice (3–5 mice per cage) were maintained in temperature-controlled rooms with a ventilation system (Alesco, Monte Mor, São Paulo, Brazil), 12 h light/dark cycle, food and water ad libitum, and suitable environment enrichment. All mice were treated according to animal welfare guidelines of the ICB (Ethics Protocol 009/2015) under National Legislation-11.794 Law.

### 2.2. Allergen

Ovalbumin (OVA) used in this study was depleted of the Lipopolysaccharides (LPS) activity, tested by Limulus amoebocyte lysate QCL-1000 kit (BioWhittaker, Walkersville, MD, USA), using 6–8 cycles of Triton X-114 extractions. The endotoxin level of the final OVA product (2 mg/mL) was below the detection limit of Limulus assay lysate (under 0.1 Endotoxin Units).

### 2.3. Alum Gel Preparation

The aluminum gel formulation (Al(OH)_3_) was prepared by precipitation of ammonium aluminum sulfate dodecahydrate (AlH_4_(SO_4_)_2_-12 H_2_O, Alfa Aesar, MA, USA) with an excess of 1N NaOH (Synth, SP, Brazil) (Alum). The Alum was mixed in water (Milli Q, ON, Canada) and washed five times for 15 min by centrifugation at 3000 rpm. The final precipitate was suspended again in water and the solution concentration was determined by calculating 1 mL of dry formulation.

### 2.4. Experimental Protocol

Mice were sensitized by the subcutaneous (s.c.) route with 4 µg of ovalbumin (OVA) (Sigma-Aldrich, St. Louis, MO, USA) or with OVA plus 10 µg CpG-ODN 2395 (CpG-ODN), a Class C TLR9 agonist (Invivogen San Diego, CA, USA) with Alum 1.6 mg on days 0 and 7. In experiments involving BM-DCs, the cells were treated with OVA or OVA plus CpG-ODN, and mice were sensitized s.c. with the cells on days 0 and 7. All mice received an intranasal (i.n.) challenged with 10 µg of OVA in 40 µL of PBS on days 14 and 21. Control mice consisted of naïve non-manipulated animals. All treatment procedures were performed under anesthesia with Xylazine 100 mg/kg plus Ketamine 10 mg/kg (Rhobifarma, São Paulo, Brazil) in the intraperitoneal (i.p.) route diluted in PBS. Animals were euthanized with the use of inhaled isoflurane (Cristália, São Paulo, Brazil) 24 h after the last challenge and all samples were collected and kept at −20 degrees Celsius until analysis.

### 2.5. Generation of Bone Marrow-Derived Dendritic Cells

BM-DCs were differentiated from femurs of C57BL/6, IL-10^−/−^, or DC-MyD88^−/−^ mice. After euthanasia, the femurs were removed under sterile conditions, both ends of the femurs were cut and the femur was flushed with 5 mL of RPMI-1640 medium (Sigma-Aldrich, St. Louis, MO, USA) with 10% fetal bovine serum (FBS) (Sigma-Aldrich, St. Louis, MO, USA) using a 5 mL syringe. Cells were plated into 6-well plates at a concentration of 1 × 10^6^ cell per well and cultured at 37 °C in 5% CO_2_ from day 0 to 4 with 20 ng/mL of GM-CSF (BioLegend, San Diego, CA, USA), and on day 4, cells were further supplemented with GM-CSF [18]. On day 6, the cells were stimulated with OVA (100 μg/well) or OVA (100 ug/well) and CpG-ODN (1 ug/well). On day 7, BM-DCs were collected, washed 3 times with sterile PBS, counted with a hemocytometer and 1 × 10^6^ cells were used for the s.c. sensitization.

### 2.6. Bronchoalveolar Lavage (BAL) Fluid Collection

The BAL fluid was collected by injecting and aspirating 1 mL of cold PBS via the trachea. Total cell counts in BAL fluids were determined by using a hemocytometer (Sigma–Aldrich, Saint Louis, MO, USA). Differential cell counts in BAL fluids were determined using cytospin (Thermo Fisher Scientific, Walthan, MA, USA) and cells were stained with a stain based on Romanowsky formulation (Instant-Prov, Newprov, SP, Brazil).

### 2.7. ELISA for Cytokines

IL-5, IFN-γ, and IL-10 levels in BAL were measured by sandwich kit ELISA according to the manufacturer’s recommendation (BD Biosciences, San Jose, CA, USA). Values were calculated based on a standards curve of recombinant cytokines ran in parallel. Values are expressed as picograms per milliliter (pg/mL).

### 2.8. Lung Histopathology Analysis

Lungs were perfused with 10 mL of cold PBS through the heart and fixed in 10% PBS-formalin for 24 h. Following, lung tissues were relocated to a 70% ethanol solution until embedding in paraffin. Five-micrometer sections were stained with hematoxylin and eosin (HE) for quantification of lung inflammation. Microphotographs of the sections of stained lung were analyzed using a Nikon E1000 microscope, equipped with a camera Nikon camera, and images were analyzed using the NIS-Elements software (Nikon Instruments Inc., Tokyo, Japan). Lung inflammation score was calculated by measurement of the peribronchial cellular infiltrates area divided by the girth of the adjacent bronchial basal membrane.

### 2.9. Statistical Analyses

Statistical analyses were performed using GraphPad Prism software (V.8; GraphPad Software, San Diego, CA, USA). One-way ANOVA followed by Tukey post-test, as appropriate. Differences were considered statistically significant when *p*-value ≤ 0.05. Data represent the mean ± SEM.

## 3. Results

### 3.1. Inhibition of Th2 Allergic Immunity by CpG-ODN Is MyD88-Dependent and Associated with Increased Production of IL-10 in Lung

**Figure 1:**
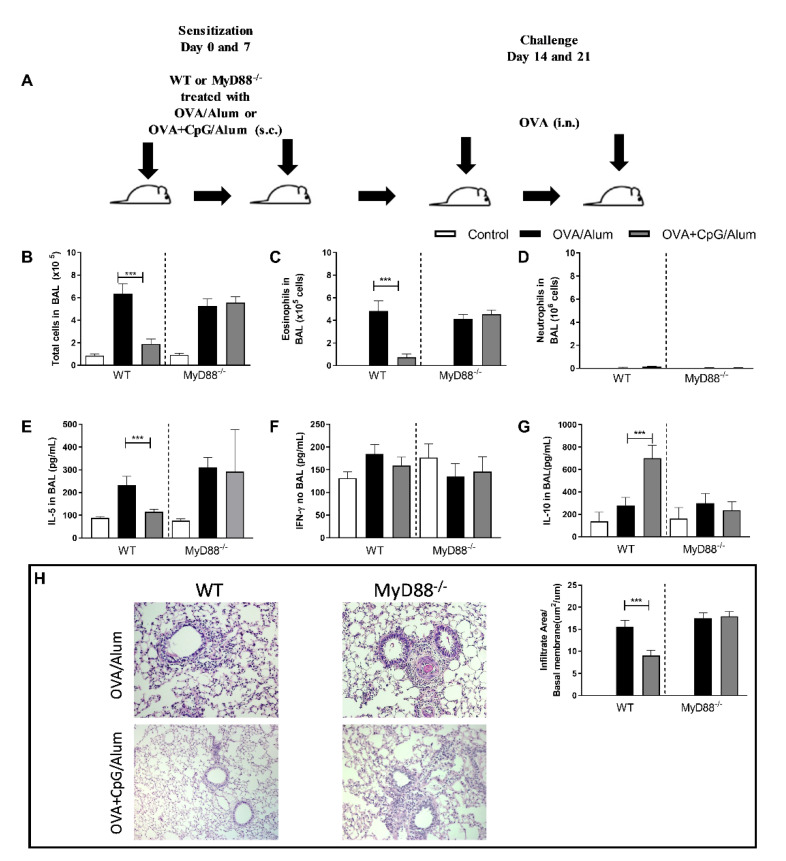
Inhibition of allergic inflammation by CpG-ODN is MyD88-dependent and associated with increased production of IL-10 in the lung. C57BL/6 wild-type (WT) or MyD88-deficient (MyD88^−/−^) were subcutaneously sensitized with alum adjuvant and ovalbumin (OVA/Alum) or OVA plus CpG (OVA+CpG/Alum) on days 0 and 7 and challenged intranasally with OVA on days 14 and 21. Experiments were performed on day 22. Control mice consisted of non-manipulated animals. (**A**) Schematic experimental protocol. Numbers of (**B**) Total Cells, (**C**) Eosinophils, and (**D**) Neutrophils in BAL. (**E**) IL-5, (**F**) IFN-γ and (**G**) IL-10 levels in BAL. (**H**) Histopathology of lung sections for hematoxylin and eosin and Lung inflammation score. Values represent the mean ± SEM and are representative of two experiments. One-way ANOVA with Tukey post-test was used, *** *p* < 0.001.

We have previously demonstrated that sensitization to allergens, with a TLR4 or TLR9 agonist, prevents the development of Th2 lung inflammation without shifting the lung inflammation towards a Th1/Th17 pattern [1,2,3]. Classically, CpG-ODN signals through the MyD88 pathway [4], but at high CpG-ODN doses, the TRIF pathway could be involved [5]. We showed that the anti-allergic effects of CpG-ODN were dependent on the MyD88 adaptor molecule [3,6]. Since CpG-ODN induces the production of IL-10, a cytokine that is known to suppress Th2 as well as Th1/17 immunity [7], we investigated whether IL-10 production could be associated with the attenuated lung allergic responses. For this, we performed experiments with WT and MyD88-deficient (MyD88^−/−^) mice to confirm the inhibitory role of MyD88 signaling on allergic sensitization (Figure 1A) and determined the levels of prototypic cytokines (IL-5, IFN-γ and IL-10) representing respectively, type 2, 1 and suppressor phenotypes. Confirming previous results, we found that the addition of CpG-ODN prevented airway allergic inflammation in WT mice but not in MyD88^−/−^ mice (Figure 1), as revealed by the reduction of the total number of cells and eosinophils in the BAL fluid in comparison to the group sensitized to OVA without CpG-ODN (Figure 1B,C), while the number of neutrophils was low in all groups and was not affected by CpG-ODN (Figure 1D). Also, WT mice sensitized with OVA+CpG/Alum showed lower IL-5 levels in BAL than the OVA/Alum group (Figure 1E), but the IFN-γ levels in BAL were similar between these groups (Figure 1F). Importantly, we found an increased IL-10 production in the CpG group compared with the control group and allergic group in WT, but not in MyD88^−/−^ mice (Figure 1G). Histopathological analysis and inflammatory score confirmed the attenuation of lung inflammation in WT mice, but not in MyD88^−/−^ mice (Figure 1H).

These results confirm and reinforce the notion that MyD88 signaling by CpG-ODN is essential for the anti-allergic effects of CpG-ODN and that the increased production of IL-10, but not IFN-γ, is associated with this inhibition.

### 3.2. MyD88-Expressing Dendritic Cells, but Not B Cells, Are Necessary for the Inhibition of Allergic Sensitization by CpG-ODN Signaling

**Figure 2:**
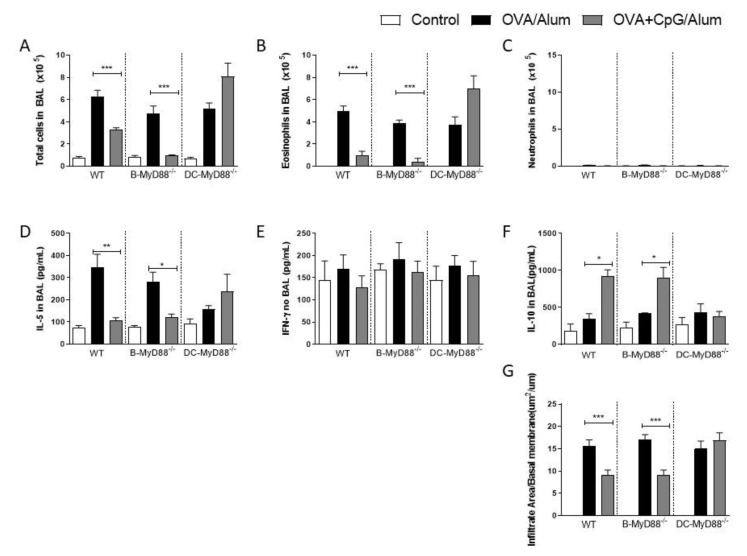
MyD88-expressing dendritic cells are necessary for the inhibition of allergic sensitization by CpG-ODN. C57BL/6 wild-type (WT) or with specific deletion of MyD88 on B cells (B-MyD88^−/−^) or with specific deletion of MyD88 on DCs (DC-MyD88^−/−^) mice were subcutaneously sensitized with alum adjuvant and ovalbumin (OVA/Alum) or OVA plus CpG (OVA+CpG/Alum) on days 0 and 7 and challenged intranasally with OVA on days 14 and 21. Experiments were performed on day 22. Control mice consisted of non-manipulated animals. Numbers of (**A**) Total Cells, (**B**) Eosinophils and (**C**) Neutrophils in BAL. (**D**) IL-5, (**E**) IFN-γ and (**F**) IL-10 levels in BAL. (**G**) Lung inflammation score in histopathology of lung sections stained with hematoxylin and eosin. Values represent the mean ± SEM and are representative of two experiments. One-way ANOVA with Tukey post-test was used. * *p* < 0.05, ** *p* < 0.01, *** *p* < 0.001.

Having confirmed the role of the MyD88 adaptor molecule, we next sought to determine whether MyD88 expressed on CD19-positive B cells or on CD11c-positive putative dendritic cells are the target cells of CpG-ODN signaling. These two cell types were selected since it is known that they produce IL-10 upon CpG-ODN stimulation [8,9,10,11,12] and are also involved in immunomodulation and allergic sensitization [13,14]. Therefore, we used mice generated by the Cre-LoxP system with specific deletion of MyD88 on B cells (B-MyD88^−/−^) or DCs (DC-MyD88^−/−^). We found that the addition of CpG-ODN during allergic sensitization effectively inhibited lung allergic responses in WT and B-MyD88^−/−^, but not on DC-MyD88^−/−^ as revealed by the decrease in the number of total cells and eosinophils in BAL (Figure 2A,B). As shown above, sensitization with CpG-ODN did not induce an increased influx of neutrophils in any group (Figure 2C). Accordingly, OVA+CpG/Alum sensitization reduced IL-5 levels in BAL in WT and B-MyD88^−/−^ when compared with their respective OVA/Alum groups (Figure 2D). IFN-γ production was similar in all groups (Figure 2E). Importantly, sensitization in the presence of CpG-ODN increased IL-10 levels in WT and B-MyD88^−/−^ mice, but not DC-MyD88^−/−^ (Figure 2F). Finally, histopathological analysis and scores confirmed that lung inflammation was reduced in WT and B-MyD88^−/−^, but not in DC-MyD88^−/−^ mice sensitized with OVA+CpG/Alum (Figure 2G).

We conclude that CpG-induced MyD88 signaling in DCs, but not in B cells, mediates the inhibition of lung inflammation, and that this inhibition is associated with increased production of IL-10 in BAL.

### 3.3. CpG-ODN in IL-10-Deficient Mice Induces Immune-Deviation towards a Th1-Dominated Airway Inflammation

**Figure 3:**
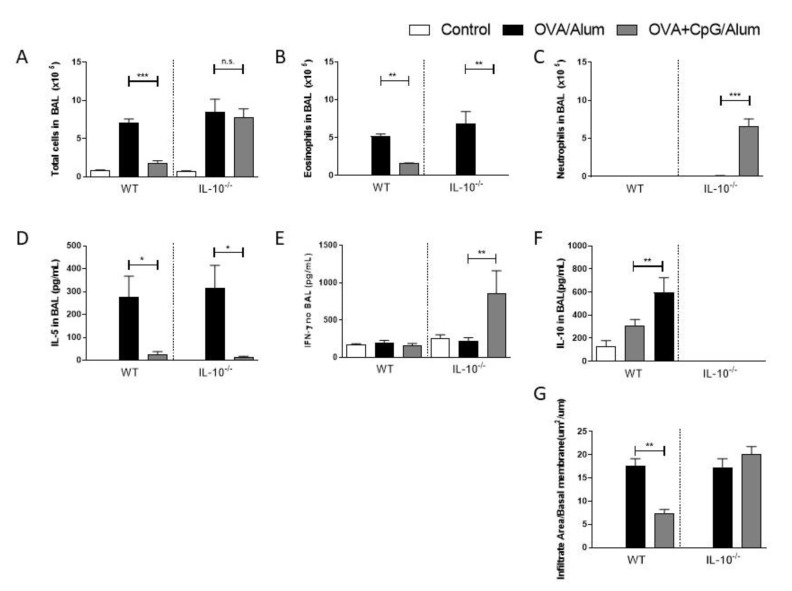
Sensitization of IL-10-deficient mice with CpG-ODN prevents allergic sensitization and induces immune-deviation towards a Th1-dominated airway inflammation. C57BL/6 wild-type (WT) or IL-10-deficient (IL-10^−/−^) were subcutaneously sensitized with alum adjuvant and ovalbumin (OVA/Alum) or OVA plus CpG (OVA+CpG/Alum) on days 0 and 7 and challenged intranasally with OVA on days 14 and 21. Experiments were performed on day 22. Control mice consisted of non-manipulated animals. Numbers of (**A**) Total Cells, (**B**) Eosinophils and (**C**) Neutrophils in BAL. Level of (**D**) IL-5, (**E**) IFN-γ, and (**F**) IL-10 in BAL. (**G**) Lung inflammation score. Values represent the mean ± SEM and are representative of two experiments. One-way ANOVA with Tukey post-test was used. * *p* < 0.05, ** *p* < 0.01, *** *p* < 0.001, n.s. = statistically non-significant.

Since increased levels of IL-10 in BAL were associated with decreased Th2-mediated responses in the airways, we next examined the effect of CpG-ODN in mice lacking IL-10. We found that IL-10-deficient mice sensitized with OVA/Alum developed an allergic lung inflammation similar to WT mice with an increased number of total cells and eosinophil in BAL when compared to control mice (Figure 3A,B), while sensitization with OVA + CpG/Alum resulted in a reduction in eosinophil inflammation in both WT and IL-10-deficient mice (Figure 3A,B). Accordingly, sensitization with CpG-ODN resulted in lower levels of IL-5 in BAL in both strains when compared with the allergic group (Figure 3D). Notably, CpG sensitization of IL-10-deficient, but not WT mice, resulted in intense neutrophilic airway inflammation (Figure 3C) with high levels of IFN-γ in BAL (Figure 3E). As expected, IL-10 levels were only present in WT mice, and treatment with CpG-ODN was associated with increased IL-10 production in BAL (Figure 3F). Histopathological analysis confirmed that CpG-ODN inhibited significant lung inflammation in WT, but not IL-10-deficient mice (Figure 3G).

These results indicate that, in the presence of IL-10, CpG-ODN inhibits Th2 immunity but does not induce Th1 immunity, while in the absence of IL-10, CpG still inhibits Th2 immunity and induces a shift towards a Th1 immunity.

### 3.4. Sensitization with Bone Marrow-Derived Dendritic Cells Lacking IL-10 and Primed with OVA and CpG-ODN Prevent Allergic Lung Inflammation but Does Not Induce Th1 Immunity

**Figure 4:**
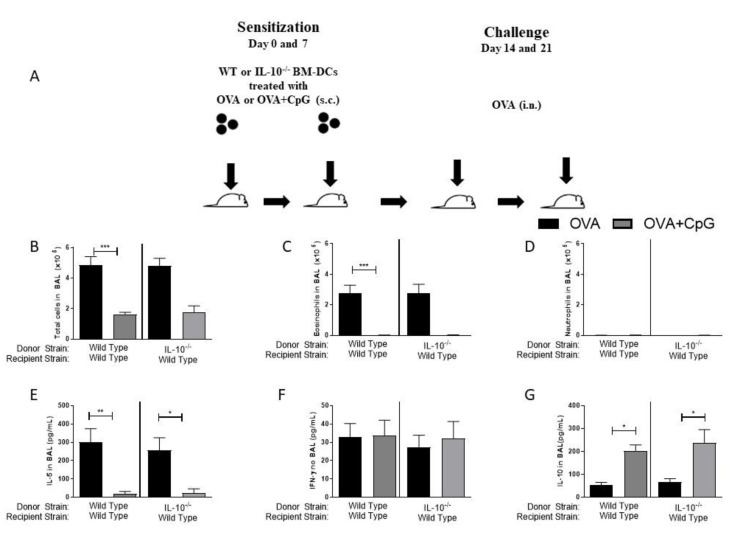
IL-10 expression on BM-DCs is not required for the anti-allergic effect of CpG-ODN. IL-10-deficient BM-DCs pulsed with OVA plus CpG-ODN inhibit allergic lung inflammation. BM-DCs generated from WT or IL-10-deficient mice were pulsed with OVA or OVA+CpG and used for the s.c. sensitization of WT mice on days 0 and 7 and challenged intranasally with OVA on days 14 and 21. Experiments were performed on day 22. Control mice consisted of non-manipulated animals. (**A**) Schematic experimental protocols. Numbers of (**B**) Total Cells, (**C**) Eosinophils and (**D**) Neutrophils in BAL. Level of (**E**) IL-5, (**F**) IFN-γ, and (**G**) IL-10 in BAL. Values represent the mean ± SEM and are representative of two experiments. One-way ANOVA with Tukey post-test was used. * *p* < 0.05, ** *p* < 0.01, *** *p* < 0.001.

Immunizations with dendritic cells (DCs) have been extensively used in immunotherapy for different purposes [13,15,16]. We decided to ascertain in our model the role of IL-10 exclusively on DCs. For this, we generated bone marrow-derived DCs from IL-10-deficient or WT mice and pulsed them with OVA or OVA+CpG. These BM-DCs were then used to sensitize WT recipients. With this type of experiment, we envisaged to determine the role of the IL-10 molecule exclusively on DCs in the anti-allergic effect of CpG as well as in the induction of Th1 immunity. We found that sensitization of WT recipients with WT or IL-10-deficient BM-DCs pulsed with OVA (Figure 4A), resulted upon allergen challenge in allergic lung inflammation characterized by an increased influx of total cells and eosinophils, but not neutrophils in the BAL (Figure 4B–D), indicating that sensitizations with BM-DCs reproduce the results obtained with allergic sensitization induced by OVA/Alum, although being less intense. Also, the addition of CpG to BM-DCs derived from WT or IL-10^−/−^ mice inhibited the development of airway allergic responses and IL-5 production upon OVA challenge (Figure 4B–E), which was associated with increased IL-10 production in BAL (Figure 4G). Sensitization with BM-DCs from IL-10^−/−^ donors did not result in neutrophilic inflammation and did not increase the production of IFN-γ in BAL (Figure 4D,F).

The results confirm directly that IL-10 molecule in DCs is dispensable for the anti-allergic effect of CpG and that the increased production of IL-10 in BAL does not derive from BM-DCs primed with CpG-ODN. Moreover, the lack of IL-10 molecule in BM-DCs was not sufficient to deviate the immune response towards a Th1 pattern indicating that CpG-ODN-induced Th1 polarization requires a more generalized IL-10 deficiency.

### 3.5. MyD88 Expression on BM-DCs Mediates the CpG-ODN Anti-Allergic Effect

**Figure 5:**
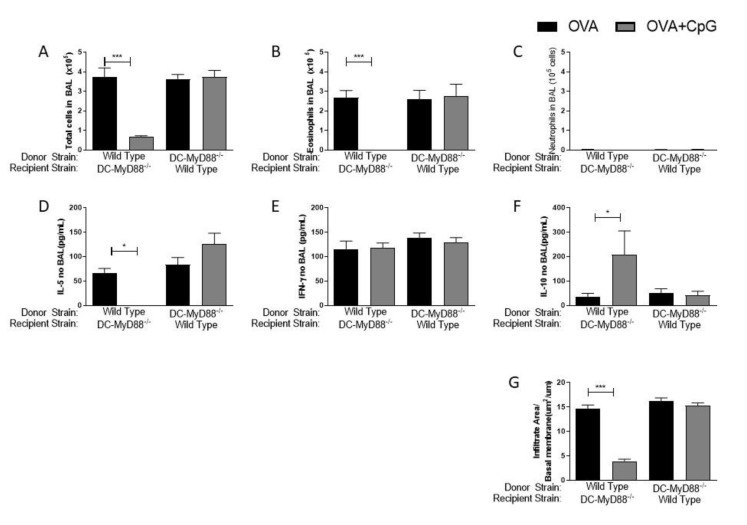
MyD88 expression on BM-DCs mediates the CpG-ODN anti-allergic effect. BM-DCs generated from WT or DC-MYD88^−/−^ mice were pulsed with OVA or OVA+CpG and used for the s.c. sensitization of DC-MYD88^−/−^ or WT mice, respectively, on days 0 and 7, and challenged intranasally with OVA on days 14 and 21. Experiments were performed on day 22. Numbers of (**A**) Total Cells, (**B**) Eosinophils and (**C**) Neutrophils in BAL. Level of (**D**) IL-5, (**E**) IFN-γ, and (**F**) IL-10 in BAL. (**G**) Lung inflammation score. Values represent the mean ± SEM and are representative of two experiments. One-way ANOVA with Tukey post-test was used. * *p* < 0.05, *** *p* < 0.001.

To further explore the role of MyD88 adaptor molecule on BM-DCs in our model of allergic lung inflammation, we generated BM-DCs from DC-MyD88^−/−^ and WT mice that were pulsed with OVA with or without CpG-ODN and transferred these BM-DCs, respectively, to WT or CD11c-positive MyD88^−/−^ recipient’s mice. As shown in Figure 5, mice sensitized with BM-DC pulsed with OVA developed an allergic lung inflammation as indicated by the increased number of total cells and eosinophils, but not neutrophils in BAL (Figure 5A–C). The allergic lung inflammation was suppressed in mice sensitized with BM-DCs pulsed with OVA and CpG from WT, but not from DC-MyD88^−/−^ (Figure 5 A,B). The number of neutrophils did not increase in CpG group and the IFN-γ levels were similar in all groups (Figure 5C,E), while IL-10 production increased in BAL of mice sensitized with BM-DCs from WT, but from DC-MyD88^−/−^ (Figure 5F). Histopathological scores confirmed that inhibition of lung inflammation required the MyD88 molecule on BM-DCs (Figure 5G).

All in all, these results demonstrate directly that MyD88-signalling on DCs is crucial for the inhibition of allergic eosinophilic airway inflammation and for the increased production of IL-10 production.

## 4. Discussion

The role of TLRs agonists and MyD88 signaling in allergic responses is quite controversial [1], exemplified by reports showing that MyD88 signaling could either promote or inhibit allergic responses [16,19,20,21,22]. We have previously shown that the MyD88 pathway is dispensable for the development of allergic lung inflammation in mice sensitized to OVA with Alum adjuvant since MyD88-deficent do develop it similarly to WT mice [15]. In contrast, we confirmed in the present work that the MyD88 pathway was crucial for the inhibitory effect of CpG-ODN on allergic responses in mice sensitized to OVA in the presence of CpG-ODN [15,23] and showed that CpG-ODN-induced inhibition of allergic inflammation was not associated with increased IFN-γ production, but was associated with increased IL-10 production in the airways. These results could indicate that IL-10 is involved in the suppression of Th1 immunity as originally described [24], as well as in the suppression of Th2 immunity as reported previously [25].

Different cell types, such as B cells, macrophages and DCs, produce IL-10 upon CpG-ODN stimulation [26,27,28]. Since B cells or DCs participate actively in allergy [29,30] and could contribute to downmodulation of Th2 immunity by the induction of B or T regulatory cells-producing IL-10 [31,32], we first determined whether MyD88 expression on these cells is required for the CpG-ODN-induced IL-10 production and consequent anti-allergic effect. Using mice with specific deletion of *Myd88* gene, we identified that DCs, but not B cells, are the key target cells for the CpG-ODN-mediated anti-allergic activity and enhanced production of IL-10. In a recent work and in line with our results, we showed that the inhibition of IgE production by CpG-ODN was also dependent on MyD88-expressing DCs and not on B cells [17].

Having established that MyD88-expressing DCs are essential for the inhibition of allergic lung responses and increased IL-10 production, we next performed experiments in IL-10^−/−^ mice. Regarding eosinophil numbers and IL-5 production in BAL, IL-10^−/−^ mice behaved similarly to WT, indicating that IL-10 is dispensable for the inhibition of allergic inflammation by CpG-ODN. However, differently to what we found in WT mice, IL-10-deficient mice developed an intense influx of neutrophils in the airways and high levels of IFN-γ in BAL, characterizing a shift towards Th1 immunity as previously reported [15].

It is known that type 1 cytokines, especially interferons, are able to dampen allergic responses [33,34,35,36]. Hence, we reasoned that the low number of eosinophils and low IL-5 production might be related to the inhibitory effect of the predominant Th1 immunity developed in IL-10-deficient mice. Since in vitro-generated BM-DCs have been employed to modulate allergic airway Th2 responses [37,38,39,40] as well as Th1 activities [41,42,43], we generated BM-DCs from IL-10^−/−^ or WT mice to address more directly the role of IL-10 on DCs. We found that sensitizations with BM-DCs from WT or IL-10^−/−^ mice primed with OVA could induce allergic lung inflammation after OVA challenge and that the addition of CpG-ODN prevented the development of allergic responses. These results indicated that IL-10-derived from DCs is dispensable for the inhibition of allergic sensitization. We also found that sensitization with BM-DC primed with OVA and CpG-ODN from IL-10^−/−^ or WT mice resulted in increased production of IL-10 in BAL, denoting that the source of IL-10 is not derived from the transferred BM-DCs. Notably, sensitization with BM-DCs primed with OVA and CpG-ODN generated from IL-10^−/−^ mice were not able to induce a Th1 shift, indicating that IL-10 deficiency solely on DCs is not sufficient to drive Th1 development. Probably a lack of IL-10 in other cell types contributes to development of Th1 immunity. This is important information for the purpose of employing DCs in tumor immunotherapy [14,44].

A possible caveat of our work is the fact that TLR9 expression in mice is found in both cDCs and plasmocytoid DCs [45], while in human, TLR9 expression is absent in cDCs [46]. However, we speculate that CpG-ODN could act in humans by stimulating monocyte-derived DCs (mo-DCs) that do express TLR9 and that, upon stimuli, produce IFN type I [47]. Alternatively, CpG-ODN could act in humans through pDCs that are known to cross-prime naive CD8 T cells by transferring antigen to cDCs through exosomes [48].

Finally, to directly ascertain the anti-allergic effect of CpG-ODN of MyD88 pathway in DCs, we performed sensitization with MyD88-deficient BM-DCs. Our results clearly showed that MyD88 signaling by CpG-ODN in DCs is necessary and sufficient for the prevention of allergic lung inflammation and enhanced production of IL-10. As discussed above, it is plausible that mo-DCs or pDCs pulsed with allergen and CpG-ODN could be used as a therapeutic tool for treatment of allergic disorders in humans.

In summary, prevention of allergic sensitization requires MyD88-expressing, but not IL-10-producing DCs.

## 5. Conclusions

Collectively, our work highlights the role of CpG-ODN in preventing allergic sensitization through DCs-expressing MyD88, but not IL-10. Our work also launches the possibility of employing DC-based vaccines or therapies against allergic disorders.

## Data Availability

Data are contained within the article.

## References

[1] Zakeri A., Russo M. (2018). Dual role of toll-like receptors in human and experimental asthma models. Front. Immunol..

[2] Karacs J., Reithofer M., Kitzmüller C., Kraller M., Schmalz S., Bleichert S., Huppa J.B., Stockinger H., Bohle B., Jahn-Schmid B. (2021). Adjuvants and Vaccines Used in Allergen-Specific Immunotherapy Induce Neutrophil Extracellular Traps. Vaccines.

[3] Shirota H., Klinman D.M. (2017). CpG Oligodeoxynucleotides as Adjuvants for Clinical Use. Immunopotentiators in Modern Vaccines.

[4] Casella C.R., Mitchell T.C. (2008). Putting endotoxin to work for us: Monophosphoryl lipid a as a safe and effective vaccine adjuvant. Cell. Mol. Life Sci..

[5] Steinhagen F., Kinjo T., Bode C., Klinman D.M. (2011). TLR-based immune adjuvants. Vaccine.

[6] Hyer R., McGuire D.K., Xing B., Jackson S., Janssen R. (2018). Safety of a two-dose investigational hepatitis B vaccine, HBsAg-1018, using a toll-like receptor 9 agonist adjuvant in adults. Vaccine.

[7] Moser M., Murphy K.M. (2000). Dendritic cell regulation of TH1-TH2 development. Nat. Immunol..

[8] Lambrecht B.N., Hammad H. (2012). Lung dendritic cells in respiratory viral infection and asthma: From protection to immunopathology. Annu. Rev. Immunol..

[9] Esterházy D., Loschko J., London M., Jove V., Oliveira T.Y., Mucida D. (2016). Classical dendritic cells are required for dietary antigen-mediated induction of peripheral T reg cells and tolerance. Nat. Immunol..

[10] Kowalewicz-Kulbat M., Szpakowski P., Krawczyk K.T., Kowalski M.L., Kosinski S., Biet F., Rudnicka W., Locht C. (2021). Decrease of il-5 production by naive t cells cocultured with il-18-producing bcg-pulsed dendritic cells from patients allergic to house dust mite. Vaccines.

[11] Jay D.C., Nadeau K.C. (2014). Immune Mechanisms of Sublingual Immunotherapy. Curr. Allergy Asthma Rep..

[12] Samarasinghe R., Tailor P., Tamura T., Kaisho T., Akira S., Ozato K. (2006). Induction of an anti-inflammatory cytokine, IL-10, in dendritic cells after toll-like receptor signaling. J. Interf. Cytokine Res..

[13] Gavett S.H., O’Hearn D.J., Li X., Huang S.K., Finkelman F.D., Wills-Karp M. (1995). Interleukin 12 inhibits antigen-induced airway hyperresponsiveness, inflammation, and Th2 cytokine expression in mice. J. Exp. Med..

[14] Vicari A.P., Chiodoni C., Vaure C., Aït-Yahia S., Dercamp C., Matsos F., Reynard O., Taverne C., Merle P., Colombo M.P. (2002). Reversal of Tumor-induced Dendritic Cell Paralysis by CpG Immunostimulatory Oligonucleotide and Anti–Interleukin 10 Receptor Antibody. J. Exp. Med..

[15] Mirotti L., Custódio R.W.A., Gomes E., Rammauro F., de Araujo E.F., Calich V.L.G., Russo M. (2017). CpG-ODN shapes alum adjuvant activity signaling Via MyD88 and IL-10. Front. Immunol..

[16] Nunes F.P.B., Alberca-Custódio R.W., Gomes E., Fonseca D.M., Yokoyama N.H., Labrada A., Russo M. (2019). TLR9 agonist adsorbed to alum adjuvant prevents asthma-like responses induced by Blomia tropicalis mite extract. J. Leukoc. Biol..

[17] Custodio R.W.A., Mirotti L., Gomes E., Nunes F.P.B., Vieira R.S., Graça L., Almeida R.R., Câmara N.O.S., Russo M. (2019). Dendritic Cells Expressing MyD88 Molecule Are Necessary and Sufficient for CpG-Mediated Inhibition of IgE Production In Vivo. Cells.

[18] Wang W., Li J., Wu K., Azhati B., Rexiati M. (2016). Culture and Identification of Mouse Bone Marrow-Derived Dendritic Cells and Their Capability to Induce T Lymphocyte Proliferation. Med. Sci. Monit..

[19] Besnard A.G., Guillou N., Tschopp J., Erard F., Couillin I., Iwakura Y., Quesniaux V., Ryffel B., Togbe D. (2011). NLRP3 inflammasome is required in murine asthma in the absence of aluminum adjuvant. Allergy Eur. J. Allergy Clin. Immunol..

[20] Thomas S.Y., Whitehead G.S., Takaku M., Ward J.M., Xu X., Nakano K., Lyons-Cohen M.R., Nakano H., Gowdy K.M., Wade P.A. (2018). MyD88-dependent dendritic and epithelial cell crosstalk orchestrates immune responses to allergens. Mucosal Immunol..

[21] Eisenbarth S.C., Piggott D.A., Huleatt J.W., Visintin I., Herrick C.A., Bottomly K. (2002). Lipopolysaccharide-enhanced, Toll-like Receptor 4–dependent T Helper Cell Type 2 Responses to Inhaled Antigen. J. Exp. Med..

[22] Eisenbarth S.C. (2008). Use and limitations of alum-based models of allergy. Clin. Exp. Allergy.

[23] Fonseca D.M., Wowk P.F., Paula M.O., Gembre A.F., Baruffi M.D., Fermino M.L., Turato W.M., Campos L.W., Silva C.L., Ramos S.G. (2015). Requirement of MyD88 and Fas pathways for the efficacy of allergen-free immunotherapy. Allergy Eur. J. Allergy Clin. Immunol..

[24] Fiorentino D.F., Bond M.W., Mosmann T.R. (1989). Two types of mouse t helper cell: IV. Th2 clones secrete a factor that inhibits cytokine production by Thl clones. J. Exp. Med..

[25] Zuany-Amorim C., Hailé S., Leduc D., Dumarey C., Huerre M., Vargaftig B.B., Pretolani M. (1995). Interleukin-10 inhibits antigen-induced cellular recruitment into the airways of sensitized mice. J. Clin. Investig..

[26] Waibler Z., Anzaghe M., Konur A., Akira S., Müller W., Kalinke U. (2008). Excessive CpG 1668 stimulation triggers IL-10 production by cDC that inhibits IFN-α responses by pDC. Eur. J. Immunol..

[27] Schmitt H., Ulmschneider J., Billmeier U., Vieth M., Scarozza P., Sonnewald S., Reid S., Atreya I., Rath T., Zundler S. (2020). The TLR9 Agonist Cobitolimod Induces IL10-Producing Wound Healing Macrophages and Regulatory T Cells in Ulcerative Colitis. J. Crohns Colitis.

[28] Bouaziz J.D., Calbo S., Maho-Vaillant M., Saussine A., Bagot M., Bensussan A., Musette P. (2010). IL-10 produced by activated human B cells regulates CD4+ T-cell activation in vitro. Eur. J. Immunol..

[29] Satitsuksanoa P., Daanje M., Akdis M., Boyd S.D., van de Veen W. (2020). Biology and dynamics of B cells in the context of IgE-mediated food allergy. Allergy Eur. J. Allergy Clin. Immunol..

[30] Humeniuk P., Dubiela P., Hoffmann-Sommergruber K. (2017). Dendritic cells and their role in allergy: Uptake, proteolytic processing and presentation of allergens. Int. J. Mol. Sci..

[31] van de Veen W., Stanic B., Wirz O.F., Jansen K., Globinska A., Akdis M. (2016). Role of regulatory B cells in immune tolerance to allergens and beyond. J. Allergy Clin. Immunol..

[32] Kushwah R., Hu J. (2011). Role of dendritic cells in the induction of regulatory T cells. Cell Biosci..

[33] Yoshida M., Leigh R., Matsumoto K., Wattie J., Ellis R., O’Byrne P.M., Inman M.D. (2002). Effect of interferon-γ on allergic airway responses in interferon-γ-deficient mice. Am. J. Respir. Crit. Care Med..

[34] Nakajima H., Iwamoto I., Yoshida S. (1993). Aerosolized recombinant interferon-gamma prevents antigen-induced eosinophil recruitment in mouse trachea. Am. Rev. Respir. Dis..

[35] Lack G., Bradley K.L., Hamelmann E., Renz H., Loader J., Leung D.Y., Larsen G., Gelfand E.W. (1996). Nebulized IFN-gamma inhibits the development of secondary allergic responses in mice. J. Immunol..

[36] Iwamoto I., Nakajima H., Endo H., Yoshida S. (1993). Interferon γ regulates antigen-induced eosinophil recruitment into the mouse airways by inhibiting the infiltration of CD4+ T cells. J. Exp. Med..

[37] Koya T., Matsuda H., Matsubara S., Miyahara N., Dakhama A., Takeda K., Gelfand E.W. (2009). Differential effects of dendritic cell transfer on airway hyperresponsiveness and inflammation. Am. J. Respir. Cell Mol. Biol..

[38] Trujillo-Vargas C.M., Ramirez-Pineda J.R., Palmetshofer A., Grunewald S., Moll H., Berberich C., Erb K.J. (2005). Mice vaccinated with allergen-pulsed myeloid dendritic cells are not protected from developing allergen-induced Th2 responses. Int. Arch. Allergy Immunol..

[39] Xu K., Wu N., Min Z., Li Z., Zhu T., Liu C., Zeng Y., Song J., Mao R., Ji H. (2020). Adoptive transfer of bone marrow-derived dendritic cells (BMDCs) alleviates OVA-induced allergic airway inflammation in asthmatic mice. Sci. Rep..

[40] Min Z., Zeng Y., Zhu T., Cui B., Mao R., Jin M., Chen Z. (2021). Lipopolysaccharide-Activated Bone Marrow-Derived Dendritic Cells Suppress Allergic Airway Inflammation by Ameliorating the Immune Microenvironment. Front. Immunol..

[41] Bagaev A., Pichugin A., Nelson E.L., Agadjanyan M.G., Ghochikyan A., Ataullakhanov R.I. (2018). Anticancer Mechanisms in Two Murine Bone Marrow–Derived Dendritic Cell Subsets Activated with TLR4 Agonists. J. Immunol..

[42] Ren S., Wang Q., Zhang Y., Song Y., Dong X., Zhang W., Qin X., Liu M., Yu T. (2018). Imiquimod enhances the potency of an exogenous BM-DC based vaccine against mouse melanoma. Int. Immunopharmacol..

[43] Matisz C.E., Faz-López B., Thomson E., Al Rajabi A., Lopes F., Terrazas L.I., Wang A., Sharkey K.A., McKay D.M. (2017). Suppression of colitis by adoptive transfer of helminth antigentreated dendritic cells requires interleukin-4 receptor-α signaling. Sci. Rep..

[44] Alcaide E.G., Krishnarajah S., Junker F. (2021). Dendritic Cell Tumor Vaccination via Fc Gamma Receptor Targeting: Lessons Learned from Pre-Clinical and Translational Studies. Vaccines.

[45] Rehli M. (2002). Of mice and men: Species variations of Toll-like receptor expression. Trends Immunol..

[46] Hornung V., Rothenfusser S., Britsch S., Krug A., Jahrsdörfer B., Giese T., Endres S., Hartmann G. (2002). Quantitative Expression of Toll-Like Receptor 1–10 mRNA in Cellular Subsets of Human Peripheral Blood Mononuclear Cells and Sensitivity to CpG Oligodeoxynucleotides. J. Immunol..

[47] Hoene V., Peiser M., Wanner R. (2006). Human monocyte-derived dendritic cells express TLR9 and react directly to the CpG-A oligonucleotide D19. J. Leukoc. Biol..

[48] Fu C., Peng P., Loschko J., Feng L., Pham P., Cui W., Lee K.P., Krug A.B., Jiang A. (2020). Plasmacytoid dendritic cells cross-prime naive CD8 T cells by transferring antigen to conventional dendritic cells through exosomes. Proc. Natl. Acad. Sci. USA.

